# A New Approach to Monitoring Urban Built-Up Areas in Kunming and Yuxi from 2012 to 2021: Promoting Healthy Urban Development and Efficient Governance

**DOI:** 10.3390/ijerph191912198

**Published:** 2022-09-26

**Authors:** Jun Zhang, Xue Zhang, Xueping Tan, Xiaodie Yuan

**Affiliations:** 1 School of Architecture and Planning, Yunnan University, Kunming 650031, China; 2 School of Geography and Planning, Sun Yat-sen University, Guangzhou 510275, China

**Keywords:** nighttime light, POI, urban built-up area, urban planning, efficient governance

## Abstract

With the rapid expansion of urban built-up areas in recent years, accurate and long time series monitoring of urban built-up areas is of great significance for healthy urban development and efficient governance. As the basic carrier of urban activities, the accurate monitoring of urban built-up areas can also assist in the formulation of urban planning. Previous studies on urban built-up areas mainly focus on the analysis of a single time section, which makes the extraction results exist with a certain degree of contingency. In this study, a U-net is used to extract and monitor urban built-up areas in the Kunming and Yuxi area from 2012 to 2021 based on nighttime light data and POI_NTL (Point of Interest_Nighttime light) data. The results show that the highest accuracy of single nighttime light (NTL) data extraction was 89.31%, and that of POI_NTL data extraction was 95.31%, which indicates that data fusion effectively improves the accuracy of built-up area extraction. Additionally, the comparative analysis of the results of built-up areas and the actual development of the city shows that NTL data is more susceptible to urban emergencies in the extraction of urban built-up areas, and POI (Point of interest) data is subject to the level of technology and service available in the region, while the combination of the two can avoid the occasional impact of single data as much as possible. This study deeply analyzes the results of extracting urban built-up areas from different data in different periods and obtains the feasible method for the long time sequence monitoring of urban built-up areas, which has important theoretical and practical significance for the formulation of long-term urban planning and the current high-quality urban development.

## 1. Introduction

Macroscopically, the urban built-up area is the area actually built within the urban administrative area, while microscopically, it is the urban construction land with the urban area as the center, including the suburbs [1]. Urban built-up area is closely related to various urban activities, which can directly measure the urbanization level of a city [2]. At present, China’s urbanization process is still progressing steadily and will continue to until around 2030 [3]. With the continuous expansion of urban built-up areas, social problems such as land use contradictions, excessive economic development differences and increasingly complex population components have become more severe, which further increases the difficulty of urban and social governance [4,5]. Additionally, the centralized construction of urban built-up areas also leads to a series of ecological problems, such as the intensification of ecological risks, etc. [6]. Therefore, the accurate identification of the current situation of urban built-up areas and the judgment of their development trend plays an important role in current urban planning and social governance.

At present, although most of the data on urban built-up areas is derived from local statistical yearbooks and government bulletins, some local governments might exaggerate the area of urban built-up areas to obtain more financial support, which makes the data more subjective and less reliable [7,8]. Additionally, the local statistical yearbook is updated slowly, which makes the real-time monitoring of urban built-up areas and the accurate judgment of development trends particularly important [9]. The main reasons are as follows: on the one hand, the current development status of the urban area can be judged through the real-time extraction of urban built-up area data. On the other hand, by observing and analyzing the changes in urban built-up areas in different periods, the development trend of the city can be evaluated, and then effective suggestions conducive to urban planning and governance can be put forward.

In the monitoring of urban built-up areas, the results of built-up areas extracted from different data are quite different [10,11]. At present, among the extraction methods of urban built-up areas, image classification based on remote sensing data is generally considered to be a feasible method for fast extraction of urban built-up areas [12]. As one of the remote sensing image data, NTL data mainly reflect the urban construction in different areas by capturing the light brightness inside the urban area at night by sensors carried by satellites. At present, NTL data has been widely used in the study of urban economy, population, regional development, and other fields [13,14,15,16]. In the study of extracting urban built-up areas based on the difference in light brightness from NTL data, selecting different lighting thresholds is the key to extracting urban built-up areas. At present, the more original and accurate methods are the dichotomy method and the fixed threshold method [17,18], but these two methods need to divide and extract NTL data on the basis of being familiar with the built-up area of the study area, resulting in poor popularity and promotion value [19]. While, in recent years, with the development of science and technology, the object-oriented method provided by eCongnition (Trmible Company, Sunnyvale, CA, USA) and other software has become the mainstream of remote sensing image feature extraction. The image semantic segmentation algorithm of the object-oriented method can learn spectral-spatial feature differences in nighttime light images to enhance the ability of information extraction [20,21,22]. However, in the study of extracting urban built-up areas with NTL data, it is found that NTL data has certain deficiencies as well, including the spillover effect of light, which leads to the extracted built-up areas being larger than the actual built-up areas. Additionally, the sole use of NTL data gradually cannot reflect the diversity of urban spaces [23,24].

In recent years, big data has been fused with NTL data in the hope of improving the problems associated with NTL, thus improving the observation accuracy of NTL in urban spaces. The commonly used big data includes POI data, mobile phone signaling data, and population migration data, etc. [25]. Compared with traditional data, these big data have the advantages of a shorter update cycle, faster update speed and wider coverage, which makes big data more advantageous than traditional data in the related studies of urban space [26,27]. As a kind of point data based on geographical location, POI data is the location collection of all geographical entities within the urban area in virtual geographic space. There is a significant correlation between POI data and NTL data in space [28,29], so researchers have tried to extract the urban built-up area by fusing POI data and NTL data. The obtained study results showed that the urban built-up area extracted by fusing POI data is more accurate [30,31]. Therefore, a considerable number of studies have been carried out on the fusion of NTL data and POI data, and good results have been obtained [32]. However, the current studies on data fusion focus too much on the single time level, so the high accuracy of the study may be contingent to some extent, and the results have not highlighted the value of urban long-term planning [33,34,35].

The extraction of urban built-up areas from single NTL data or the fused NTL with POI data is essentially the extraction of image information with features of built-up areas [36], which makes deep learning and other artificial intelligence methods more advantageous. Involving many disciplines, such as the theory of probability, statistics, approximation theory, convex analysis, and algorithm complexity theory, deep learning constantly improves its own performance by studying computer simulation and realizing human learning behavior [37], which makes it possible to strengthen the interpretation of image information by constantly learning the rules and features of sample data in the process of image information feature extraction. Therefore, the method based on deep learning has more obvious advantages in image information extraction [38,39]. Some researchers have achieved higher classification accuracy by supervised classification of remote sensing images by using support vector machines, and some researchers have achieved image feature extraction through the semantic segmentation of images using fully convolutional networks [40,41].

At present, no matter what kind of data and methods are used to study the urban built-up areas, most of them stay in a single time section [42]. Although the verification results also have high accuracy, the urban built-up areas are constantly changing dynamically. Moreover, the results of different methods and data in extracting the urban built-up areas in a single time section may be accidental [43]. For example, is there any difference in the accuracy of the urban built-up areas extracted in different years? Secondly, discontinuous built-up areas play a very limited role in urban and rural planning and governance, and in the actual process, it is often necessary to extract urban built-up areas at different times and places. Therefore, based on the monitoring of the changes in urban built-up areas in Kunming and Yuxi in the past decade, this study verifies the reliability of extracting urban built-up areas through single NTL data and POI_NTL data under the method of deep learning and summarizes a set of multi-period urban built-up area extraction methods and processes with promotion value judged by the accuracy of the extraction of urban built-up areas in different periods, so as to put forward feasible suggestions for urban and rural planning and construction and urban governance in different cities.

The phases of this study are as follows: first, the data and methods involved in the extraction of built-up areas are reviewed. Second, the Landsat, NTL and POI data needed for the study are acquired and preprocessed. Third, the study methods are introduced, including image fusion, image segmentation and accuracy verification. Fourth, the urban built-up area is monitored based on NTL data. Fifth, the urban built-up area is monitored based on the fusion of NTL data and POI data. Sixth, the accuracy verification and difference analysis of different monitoring results are carried out. In terms of section organization, Section 1 provides the literature review, Section 2 discusses the study materials and methods, Section 3 presents the study results, and Section 4 and Section 5 are the discussion and conclusion, respectively.

## 2. Materials and Methods

### 2.1. Study Area

**Figure 1:**
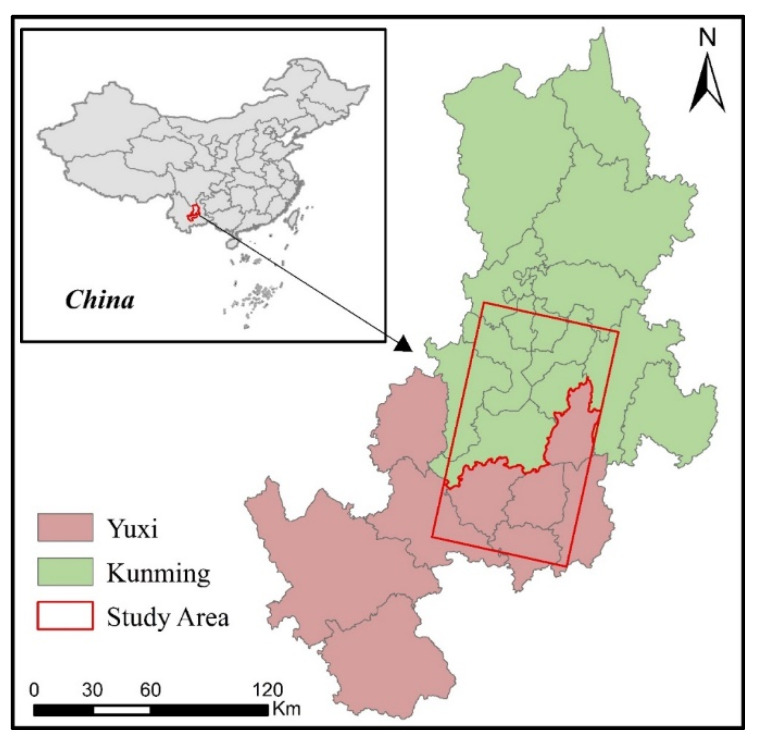
Study Area.

Kunming and Yuxi are the two fastest-growing cities in southwest China in recent years, with recorded urban GDP growth of more than 9.1% in the past ten years and an area increase of 272% and 348%, respectively, ahead of the Chinese average. Additionally, with the advancement of China’s “One Belt, One Road” initiative, Kunming and Yuxi have ushered in new opportunities for urban construction and development [44]. Therefore, this study takes Kunming and Yuxi as the study area (Figure 1).

### 2.2. Study Data

The data used in this study include the urban built-up area data, NTL data and POI data of Kunming and Yuxi from 2012 to 2021. The urban built-up area data are obtained from the official statistical yearbook, Landsat satellite image and Google Earth high-resolution image interpretation, NTL data are obtained from NPP/VIIRS, and POI data are obtained from the open API provided by Amap. The acquisition methods and attributes of different data are shown in Table 1.

Due to the subjectivity of urban built-up area data published in local statistical yearbooks, TM, ETM+ and OLI-TIRS images taken by Landsat satellite are used in this study to modify the built-up area published in statistical yearbooks through land use classification interpretation. As a land resources technology satellite launched by NASA, Landsat has been used for studies related to global agriculture, mapping, forestry, and regional planning. Landsat images can be downloaded for free on the Geospatial Data Cloud Platform (http://www.gscloud.cn/, accessed on 3 July 2022). In this study, the satellite image data from 2012 to 2021 were first downloaded, and then the multi-band remote sensing images of Kunming and Yuxi in the past ten years could be obtained by carrying out resampling, geometric correction, and image fusion. Then, through classification and interpretation, the urban construction land was obtained to correct and judge the statistical yearbook data. Finally, the urban built-up area data of Kunming and Yuxi in the past decade was obtained. Since the statistical yearbook data of the built-up area is only a numerical value, it is necessary to use the urban construction land obtained from Landsat image interpretation to correct the statistical yearbook data. The specific correction process was as follows: first, compare the area of urban construction land obtained through interpretation with the area of built-up area in the statistical yearbook, and then correct the interpretation results according to the differences obtained, so that the interpretation results are as close as possible to the actual area of the built-up area in the statistical yearbook. The final reference built-up area not only has the size of the built-up area, but also the space range. The built-up area of the Kunming and Yuxi Statistical Yearbook is shown in Table 2.

**Figure 2:**
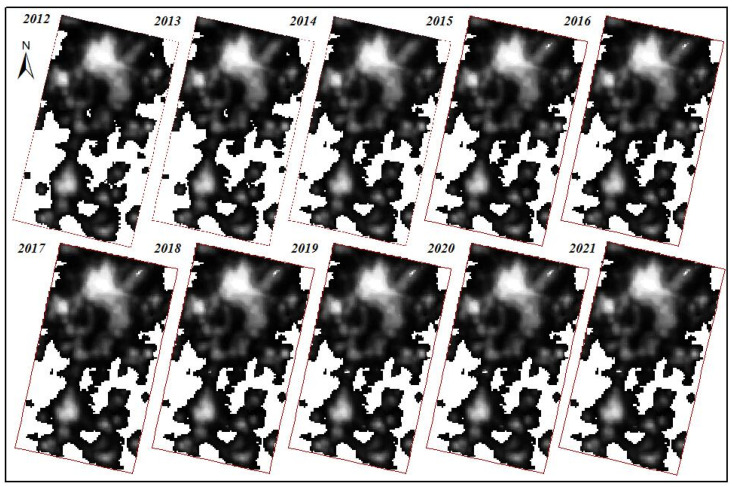
Preprocessing Results of NTL data in Kunming and Yuxi in the Past Decade.

NTL data mainly includes DMSP/OLS data and NPP/VIIRS data. The time series of DMSP/OLS data was from 1992 to 2013, and that of NPP/VIIRS data was from 2012 to 2021. Additionally, compared with DMSP/OLS data, the sensor of NPP/VIIRS has the advantages of higher clarity and higher sensitivity, and there was no obvious supersaturation phenomenon of the NPP/VIIRS NTL data. Therefore, compared with DMSP/OLS data, the NPP/VIIRS data had obvious advantages. In addition, the observation period of the NPP/VIIRS NTL data was 12 h, and the wavelength range was 505–890 μm. The faster data acquisition speed was more conducive to the calculation of monthly average and thus makes the NTL more accurate. In this study, NTL data from 2012 to 2021 was downloaded from https://eogdata.mines.edu/products/vnl/, (accessed on 24 July 2022), and then the NTL data of each year was averaged to avoid large differences in the brightness of single scene lights. Finally, the NTL data of Kunming and Yuxi from 2012 to 2021 was obtained by carrying out radiometric correction, brightness conversion and desaturation processing, as shown in Figure 2.

Although POI data can be obtained through API of Amap, Baidu and Tencent at present, the high popularity of Amap makes POI services provided by Amap more comprehensive and accurate. The attributes of the POI data obtained based on Amap API included the name, latitude, longitude, address, and category of the POI, etc. After rechecking, cleaning, and screening of the obtained POI data, the number of POI finally obtained is shown in Table 3.

### 2.3. Methods

#### 2.3.1. Kernel Density Analysis (KDA)

Kernel density analysis reflects the spatial agglomeration form of point elements by calculating the distribution of different points in geographical space [45]. The formula for kernel density analysis is:

(1) $$p_{i}=\frac{1}{n\pi R^{2}}\times \overset{n}{\underset{j=1}{\sum }}k_{j}{\left(1-\frac{D_{ij}^{2}}{R^{2}}\right)}^{2}$$

where, $p_{i}$ is the kernel density value of the spatial position, $\;D_{ij}$ is the distance between the spatial point i and the study object *j*, n is the spatial position whose distance is less than or equal to $D_{ij}^{2}$, $k_{j}$ is the spatial weight, and R is the search radius.

#### 2.3.2. Image Fusion Modification

It is increasingly difficult for a single NTL data to adapt to the drastic changes in its interior space in the study of an urban area. At present, the POI data and NTL data are fused to modify the night light by researchers. The commonly used methods of data fusion include arithmetic fusion, pyramid fusion and scale-change fusion, etc. [46,47]. As a classic multi-scale image fusion method, wavelet transform can scale data images at different scales compared with other image fusion methods, making its fusion effect far superior to other methods [48]. Wavelet transform performs multi-scale feature fusion on the data image in the horizontal, vertical, and diagonal frequency band of information through a dynamic time-frequency window. Without losing the original information of the image, the fused image can reflect the information of different data images at the same time, thus greatly improving the efficiency of using different data [49].

(2) $$WT\left(\alpha ,\tau \right)=f\left(t\right)\varphi \left(t\right)=\frac{1}{\sqrt{\alpha }}f\left(t\right)\overset{+\infty }{\underset{-\infty }{\int }}\varphi \left(\frac{t-b}{\alpha }\right)dt$$

where, $f\left(t\right)$ is the signal vector, $\varphi \left(t\right)$ is the wavelet function, α controls the scaling of the wavelet function, τ controls the translation of the wavelet function, and b is the parameter.

#### 2.3.3. Image Characteristics Extraction

The essence of extracting urban built-up areas is to extract pixel areas with built-up area characteristics from NTL data and POI data images. In the field of image feature extraction, methods such as machine learning can save data processing time and greatly improve the accuracy of processing by the use of efficient algorithms to make feature extraction simple and efficient [50]. Although convolution neural networks and full convolution neural networks are commonly used in image feature extraction based on NTL data, convolution neural networks can only input images of a fixed size when extracting image feature information, it is easy to lose some image information, and the training time for samples is relatively long [51,52]. Therefore, based on the characteristics of different algorithms in the field of machine learning, this study aims to extract the features of built-up areas by a U-net neural network, which is evolved and improved by a full convolution neural network [53]. Compared with the original FCN, the U-net neural network can comprehensively synthesize the global and local details of the image and concatenate the results of each layer of the encoder into the decoder to get better segmentation results so as to improve the accuracy of image feature extraction [54].

Equations of the Component Layers of the U-Net.

Layer Convolution:

(3) $$C_{out\;j}=bias\left(C_{out}\right)+\overset{C_{in}-1}{\underset{k=0}{\sum }}weight\left(C_{out\;j},k\right)*input\left(k\right)$$

Layer Max-pooling:

(4) $$out\left(C_{j},h,w\right)=max_{m=0,\ldots \ldots ,kH-1}max_{n=0,\ldots \ldots ,kW-1}input\left(C_{j},stride\left[0\right]\times h+m,stride\left[1\right]\times w+n\right)$$

Layer ReLU:

(5) $$ReLU\left(x\right)=max\left(0,x\right)$$

Layer softmax:

(6) $$Softmax\left(x_{i}\right)=\frac{exp\left(x_{i}\right)}{\sum_{j=1}^{k}exp\left(x_{i}\right)}$$

Layer Cross-entropy:

(7) $$loss\left(x,y\right)=-log\left(\frac{exp\left(x\left[y\right]\right)}{\sum_{j=1}^{k}exp\left(x\left[y\right]\right)}\right)$$

In Equation (3), the sizes of the input and output images are ($C_{in}$, H, W) and ($C_{out}$, $H_{out}$, $W_{out}$) where C denotes the number of channels, H is the height of the input planes in pixels, W is the width in pixels, * is the valid cross-correlation operator, and j is the j-th channel of the output feature map. In Equation (4), (kH, kW) denotes the kernel size of the pooling, and h and w refer to the height and width of the output image, respectively. In Equation (5), x denotes the pixel values of the input feature map. In Equation (6), is the i-th pixel value of the input feature map, and K is the number of classes. In Equation (7), x and y refer to the predicted and reference pixel values, respectively, and K is the number of classes [39,55].

#### 2.3.4. Accuracy Verification

In order to verify the accuracy of this study in monitoring the urban built-up areas, the following two methods were used. The first was to conduct random point sampling in the built-up area and non-built-up area of Google Earth to determine its accuracy by the Kappa consistency test. The second was to test the degree of spatial overlap between the extracted built-up area and the verified built-up area (impervious ground interpreted by land use) [56]. That is, the first verification data was the verification points randomly selected in Kunming and Yuxi area, while the second verification data was the result of the mutual correction between the urban construction land interpreted by Landsat and the built-up area of the statistical yearbook.

The formula for the consistency check is:

(8) $$k=\frac{p_{o}-p_{e}}{1-p_{e}}$$

(9) $$p_{e}=\frac{a_{1}\times b_{1}+a_{2}\times b_{2}+\ldots \ldots a_{i}\times b_{i}}{n\times n}$$

where $p_{o}$ is the overall accuracy, a is the number of real samples, b is the number of predicted samples in each class, and n is the total number of samples.

(10) $$precision=\frac{a_{overlap}}{a_{computed}}$$

(11) $$recall=\frac{a_{overlap}}{a_{comparative}}$$

(12) $$F_{1}=(\frac{2}{recall^{-1}+precision^{-1}})=2\cdot \frac{precision\cdot recall}{precision+recall}$$

where, the $F_{1}$ score is the harmonic average of recall and precision, ranging from 0 to 1, the larger the value is, the higher the precision would be, $a_{overlap}$ is the total area of the overlap between the extracted and reference built-up areas, $a_{computed}$ is the total area of the extracted built-up areas, and $a_{comparative}$ is the total area of the reference built-up area.

**Figure 3:**
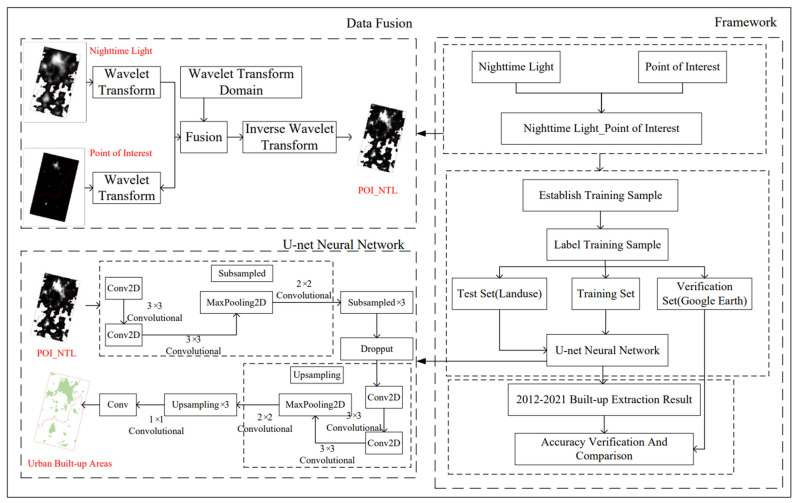
Research Process and Technical Route.

On the basis of data fusion, the study process and technical route of urban built-up area monitoring in Kunming and Yuxi in the past ten years by using the U-net method are shown in Figure 3.

Figure 3 can be divided into three parts, including data fusion, U-net neural network, and flow chart. The steps in the data fusion are that the NTL data and POI data were respectively fused by wavelet transform in the wavelet transform domain, and then the fused figure was obtained by carrying out inverse wavelet transform. While in terms of the U-net neural network, there are a total of 23 convolution layers in the U-net neural network, including 3 pixels * 3 pixels feature extraction convolution, up-sampling 2 pixels * 2 pixels convolution, and memorizing the final mapping 1 pixel * 1 pixel convolution. As for the flow chart of the study, the first step was data sorting, including NTL data, POI data and NTL_POI data obtained through the fusion of the NTL data and POI data. The second was the specific operation process of the U-net neural network, including establishing training samples, labeling training samples, and dividing training samples into test set, training set, and verification set. The last part was the extraction of the built-up areas and the verification of the results.

## 3. Results

There were obvious spatial differences between built-up areas and non-built-up areas, which mainly reflected urban geography, economic development level, and spatial structure. On the other hand, the main reason why the NTL data and POI data can both extract urban built-up areas is that there are differences in light brightness and POI data in different areas, which is caused by the difference in the regional development level of the urban area. This difference was also an important basis for the extraction of the urban built-up area of this study [57].

### 3.1. Monitoring Urban Built-Up Area Using NTL Data

Since there was a significant difference in the brightness of nighttime lights between urban built-up areas and non-built-up areas, so in the process of extracting built-up areas, NTL data could be used to identify built-up areas from non-built-up areas. According to the NTL data preprocessing results of the Kunming and Yuxi areas in the past ten years (Figure 2), it was seen that the nighttime light brightness was mainly concentrated in the Wuhua District, Guandu District, Xishan District, Panlong District, Chenggong District of Kunming, Hongta District, Jiangchuan District, and High-tech District of Yuxi. From the change in light brightness data, it was seen that there was a significant change in light brightness in 2013 and after 2019.

**Figure 4:**
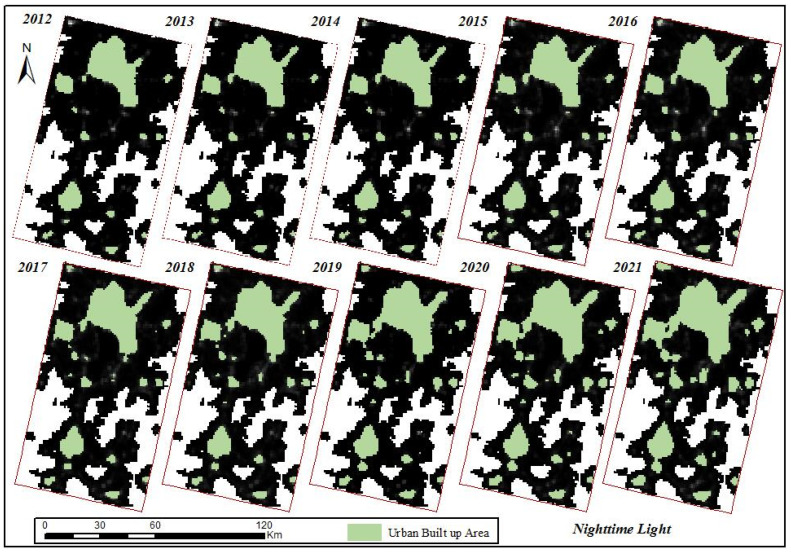
Monitoring Results of Built-up Areas in Kunming and Yuxi Area Extracted by NTL data.

By selecting the impervious ground in the classified land use data as the training sample for urban built-up areas in the Kunming and Yuxi areas, this study used a U-net neural network to extract image features from NTL data from 2012 to 2021. The built-up areas extracted in different years are shown in Figure 4. As shown in Figure 4, the built-up areas extracted from the NTL data in different years are 278.45 km^2^, 369.59 km^2^, 392.22 km^2^, 396.49 km^2^, 422.56 km^2^, 424.33 km^2^, 429.02 km^2^, 435.82 km^2^, 450.71 km^2^, and 501.91 km^2^, respectively, which accounted for 86.41%, 87.62%, 88.99%, 88.79%, 89.04%, 89.00%, 89.27%, 89.73%, 85.78%, and 84.40% of the reference built-up area, respectively. Firstly, from the perspective of the scope of the extracted built-up areas, the built-up areas were mainly concentrated in the Wuhua District, Xishan District, Panlong District, Guandu District of Kunming, and Hongta District of Yuxi, while the built-up areas extracted from the Chenggong District of Kunming and Jiangchuan District of Yuxi were less. Secondly, from the extracted built-up area patches, the built-up areas extracted before 2016 are severely fragmented with a large number of patches. While after 2016, the integrity of the patches was strengthened, especially in the built-up area of the main urban area, and the fragmented patches were mainly distributed in Chengjiang County, Tonghai County, and other small counties. Additionally, after 2016, the urban built-up areas were mainly expanded at the junction area of Kunming and Yuxi, reflecting the development of urban integration between Kunming and Yuxi. Therefore, it was found that the urban built-up areas extracted by the NTL data in the past ten years basically reflected the real urban development situation of the Kunming and Yuxi area. Even though the number of built-up areas extracted by the NTL data was enormous, the boundary was complex, and the proportion of the extracted built-up areas was not high, therefore it is necessary to further improve the extraction of built-up areas by using effective means.

### 3.2. Monitoring Urban Built-Up Area Extracted Urban Built-Up Area by Fusing NTL Data and POI Data

POI data is the virtual expression of urban entities in urban space, so the number of POI reflects the development level of different urban areas to a certain extent. In urban areas, due to the unbalanced and insufficient regional development, there are also obvious gaps in the construction of urban facilities in different regions. This gap is fed back to the POI data, which means that the degree of POI agglomeration in different regions is different. Therefore, similarly, POI data can also be used for extracting built-up areas. By analyzing the POI data in the Kunming and Yuxi area from 2012 to 2021, it was found that the POI data from 2016 to 2019 showed a sharp increasing trend, which may be caused by the rapid development of the city.

**Figure 5:**
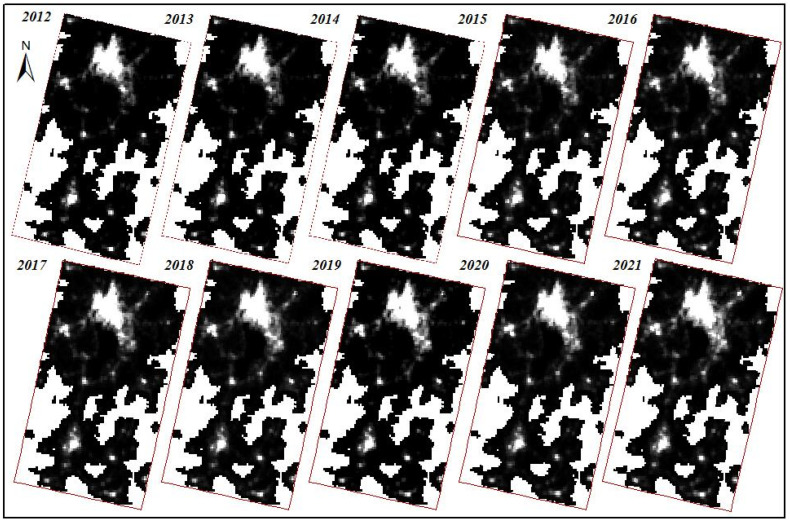
Extracted Results after NTL Data and POI Data Fusion.

POI data and NTL data were fused by wavelet transform, and the fused images of the Kunming and Yuxi area in the last ten years are shown in Figure 5.

**Figure 6:**
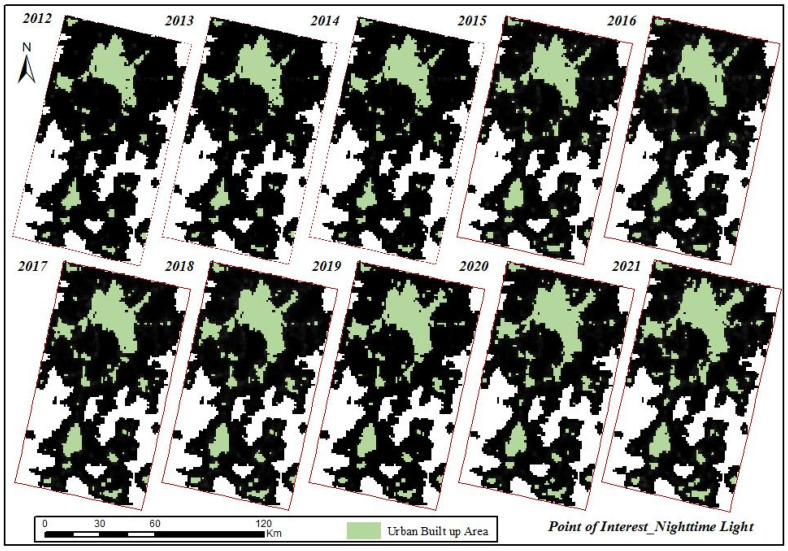
Monitoring Results of Built-up Area after Fusion of NTL data and POI data.

By using the U-net neural network to extract the built-up areas in the Kunming and Yuxi area after the fusion of the NTL data and POI data, this study extracted built-up areas in different years (Figure 6). It is shown in Figure 6 that the urban built-up areas extracted after the fusion of the POI data and NTL data were 269.33 km^2^, 358.41 km^2^, 369.98 km^2^, 378.03 km^2^, 402.34 km^2^, 432.12 km^2^, 439.28 km^2^, 451.19 km ^2^, 490.27 km^2^, and 558.42 km^2^, respectively, which accounted for 83.58%, 84.97%, 83.95%, 84.66%, 84.78%, 90.63%, 91.40%, 92.89%, 93.31%, and 93.90% of the reference built-up area, respectively. Firstly, from the perspective of the distribution range of the extracted built-up areas, the built-up areas extracted after data fusion were similar to those extracted from single NTL data, which were concentrated in the Wuhua District, Xishan District, Panlong District, Guandu District, Chenggong District of Kunming, and Hongta District of Yuxi, while the built-up areas extracted from the same period after data fusion were larger than those from the single NTL data. Secondly, from the extracted patches of built-up areas, it was seen that the fragmentation degree of the patches in urban built-up areas before 2015 were more obvious than those after 2015. Additionally, the smaller patches in the built-up areas before 2015 were mainly concentrated in Chenggong District, Chengjiang County, and Tonghai County, while after 2015, they were mainly concentrated in Chengjiang County and Tonghai County, except for Chenggong District, which was mainly because Chenggong District, as a new district of Kunming, has been vigorously developed since 2015. Therefore, the number of POI greatly increased since 2016, which was in line with the actual urban development situation in the Kunming and Yuxi areas. According to the results of urban built-up area extraction after the fusion of NTL data with POI data, it can be concluded that the area extracted was larger, and the degree of patch fragmentation was improved.

### 3.3. Accuracy Verification and Difference Analysis

In previous studies of the verification of the extraction of built-up areas, the verification is often done by sampling and verifying the reference built-up areas and non-built-up areas, but this method is more of a macroscopic judgment on the extraction of built-up areas. The judgment of the edge of the city is not necessarily accurate, let alone provide a measure of the degree of extraction refinement. Therefore, this study comprehensively judged the accuracy of the extracted built-up areas in different years through two aspects. Firstly, 3000 pixels were randomly selected from the referenced built-up area and non-built-up area in different years, among which 1000 pixels were training samples and 2000 pixels were test samples. The accuracy of the built-up area extraction was obtained by carrying out the Kappa consistency test on the successfully verified pixels in the built-up area and non-built-up area [55]. Secondly, by analyzing the spatial coincidence degree of urban built-up areas extracted from high-resolution Google Earth images from 2012 to 2021 and the urban built-up areas extracted in this study, the accuracy of the urban built-up areas outside the central urban area, especially in the urban fringe area, was obtained. The accuracies of the two verification results of extracting built-up areas are shown in Table 4.

As shown in Table 2, the accuracy of the urban built-up area extracted by the NTL data can be roughly divided into two stages. The first stage was from 2012 to 2019, when the accuracy of the built-up area extracted from the NTL data was generally above 88% and reached the highest accuracy (89.31%) in 2019. In the second stage, after 2019, the accuracy of the extraction of built-up areas showed a declining trend, with an accuracy of 85.89% in 2021. Combined with the urban development of the Kunming and Yuxi areas, after 2013, the urbanization process rapidly increased, and the urban built-up area expanded significantly. Especially after 2016, Chenggong District being one of the important construction areas, the expansion of built-up areas not only accelerated the spread of nighttime light but also affected the extraction accuracy of the built-up areas. Therefore, the extraction accuracy of the NTL data showed a slight upward trend after 2016 and reached its peak in 2019. After 2019, although the expansion of the built-up area in Kunming and Yuxi was more obvious, and the increase of the built-up area reached more than 10%, the accuracy of the NTL data extraction showed a declining trend, with an accuracy of 85.89% in 2021, which may be related to the COVID-19 epidemic. With the outbreak of COVID-19 at the end of 2019, by 2021, although the urban development and construction in Kunming and Yuxi had continued, the physical industry suffered heavy losses resulting in the stagnation of a large number of industries in some areas, especially in the urban outlying areas, further causing the reduction of light brightness at night. Additionally, the isolation policy during the epidemic in the urban center led to a certain weakening of the lights, which affected the accuracy of the NTL data extraction in the built-up areas.

Analysis of Table 2 shows that the accuracy of the urban built-up area extracted after NTL data and POI data fusion can be divided into three stages. The first stage was from 2012 to 2016, when the accuracy of extracting urban built-up areas remained stable at about 92%. Considering the urban development of Kunming and Yuxi, the reason should be the POI data. Before 2016, the POI data had been stable at a low amount. On the other hand, due to the immaturity of the technology, only some major POI data are counted before 2016. The second stage was from 2016 to 2019, when the accuracy of POI_NTL data extraction was above 95%, which has high accuracy. The possible reason is that with the strong construction of Chenggong District and the development and maturity of technical services during these years, more and more urban infrastructure was counted into POI. The number of POI greatly increased, which ensured the accuracy of extraction of built-up areas. The third stage was from 2020 to 2021, where the accuracy of POI_NTL extraction decreased to about 93%. This may be due to the impact of the COVID-19 epidemic, which led to the disappearance of some POI services. Moreover, POI_NTL data is affected in the process of extracting built-up areas due to the reduced brightness of light at night.

In general, the NTL data and POI_NTL data both played an important role in the study of extracting urban built-up areas from 2012 to 2021 and accurately reflected the actual situation of local urban development, especially the nodes of urban development and construction and urban public emergencies. This makes NTL data and POI_NTL data have higher reference values in extracting urban built-up areas. However, judging from the extraction results of the two kinds of data, it was found that although the accuracy of urban built-up areas extracted from the NTL data was more stable, single NTL data was easily affected by public health emergencies, such as COVID-19. The sudden public health emergencies can cause the spatial distribution of light brightness to be quite different from the past, which affects the accuracy of extraction. On the other hand, POI data are easily affected by technical services. In immature and remote areas, the statistics of POI data are often not accurate enough, which seriously affects the accuracy of POI_NTL data extraction in built-up areas. Therefore, it can be concluded that the two types of data have their own characteristics and advantages. From the results of the two types of data, the NTL data is more suitable for extracting built-up areas in long-term series and without urban emergencies, while the POI data is more suitable for technical and service mature areas to carry out monitoring of built-up areas. In general, the effect of extracting built-up areas from a single NTL data is not as good as that of the fused POI_NTL data. Although the fusion of NTL data and POI data significantly improves the extraction accuracy of the built-up area, the fusion includes not only their advantages but also their shortcomings, which was illustrated by the three different stages of the POI_NTL data extraction results.

## 4. Discussion

In the previous studies, the statistical data of urban built-up areas mainly originates from the data published by local governments and the data extracted by remote sensing studies. However, the data of built-up areas published by local governments are often overstated, while the accuracy of urban built-up areas extracted by remote sensing data is generally around 85% [20,58]. At present, although relevant scholars have developed many built-up area extraction systems based on NTL data and POI data, the extracted built-up areas usually only have a single time section, while the characteristics and effects of different data have not been explained in the context of a long time series [59]. Therefore, there is a general lack of scientific, objective, and unified methods to extract urban built-up areas, especially for monitoring built-up areas over a long time sequence [60]. Based on this, NTL data and POI_NTL data were used to extract the characteristics of urban built-up areas through deep learning methods in the ten years from 2012 to 2021. The results of the extracted built-up areas were analyzed by various methods. It was hoped that the characteristics and advantages of different data in extracting urban built-up areas could be obtained, and then a feasible built-up area monitoring system could be summarized to provide a feasible reference for urban planning and management.

As a kind of remote sensing data, NTL data has played an important role in related studies on the extraction of urban built-up areas [61]. However, with the rapid expansion of urban areas and the acceleration of urbanization construction in rural areas, it has gradually become difficult to distinguish the characteristics of built-up areas and non-built-up areas using single NTL data, and new data methods need to be supplemented to improve the extraction accuracy of urban built-up areas [62,63,64]. At present, based on the high correlation between NTL data and POI data in urban space, the urban built-up area is extracted by fusing NTL data and POI data, and the modification effect of NTL data is achieved by introducing POI data [65]. However, the current studies on the fusion of different data, including POI and NTL data, to extract urban built-up areas focus on a single time section, while there is a certain degree of contingency reflected by the single time section, it does not necessarily fully explain the promotion value of extracting urban built-up areas by fusing different data using this method. Based on the fusion of NTL data and POI data [66], this study makes a more convincing conclusion through the extraction results and accuracy analysis of urban built-up areas in the decade from 2012 to 2021 on the actual urban development of in the Kunming and Yuxi areas.

The extraction of urban built-up areas basically depends on the data used. From the remote sensing data to the fusion with big data, the methods of extracting built-up areas have gradually shifted from the threshold method and dichotomy to image segmentation and feature extraction [67,68]. The results of the built-up areas extracted based on these methods are also different, but these methods are difficult to generalize to long time sequences [69]. Starting from image feature extraction, this study further introduces deep learning to extract urban built-up areas. Although the cost of feature training is high, the ease of promotion and high accuracy make this study method more valuable [70]. By analyzing and comparing other studies on big data fusion extraction of urban built-up areas in recent years, it can be found that the deep learning method used in this study can effectively avoid the misjudgment of foreign bodies with the same spectrum and different spectra of the same objects compared with remote sensing image extraction and improve the model structure and classification accuracy compared with other machine learning model methods [71,72]. Overall, the deep learning method used in this study has unique advantages in operability, accuracy, and generalizable replication compared with other methods, which can greatly enrich the extraction and monitoring of urban built-up areas.

Compared with the extraction studies of other urban built-up areas, the main innovation of this study was firstly the use of the deep learning method to monitor the changes in built-up areas in Kunming and Yuxi from 2012 to 2021 based on the fusion of NTL data and POI data. Next, this study analyzed and tested the characteristics and deficiencies of NTL data and POI data in extracting urban built-up areas over a long time sequence, which has not been considered in previous studies. Then this study obtained a relatively reliable and feasible method for the continuous monitoring of urban built-up areas. Secondly, the use of a deep learning method to extract urban built-up areas also promoted the theoretical research of urban space. Although this study takes the Kunming and Yuxi area as a case point for analysis, the results obtained in this study show that it is feasible to use the U-net to observe long time series of built-up areas on the basis of NTL data and POI data. However, due to certain differences in the development of different cities and regions, the results of this study cannot be extended to other cities without change. Even so, this study still has a certain reference value in terms of ideas and methods for the observation of built-up areas in different cities over a long time series.

However, there are still some shortcomings in this study, mainly because the data used in this study include NTL data and POI data, which are both static data in space. Some studies have shown that static data may not necessarily reflect the most real urban built-up areas. At present, the latest studies have fused dynamic data, including big data on population migration, with static data to extract built-up areas and have achieved good results [49]. However, due to the lack of a time series for population migration data in the past ten years, this study does not provide supplementary analysis. In the future, we will further explore the combination of possible dynamic data with NTL data and POI data to monitor the changes more accurately in built-up areas over a long time series, which is of great significance for urban planning and management.

## 5. Conclusions

As one of the manifestations that can directly reflect urban development, the ability to accurately monitor the dynamic changes in urban built-up areas in real-time is of great value for urban planning and management. In this study, the highest built-up area extraction accuracy using a deep learning U-net neural network to extract features of NTL data was 89.31%, and that of POI_NTL data was 95.31%, indicating that the U-net method on the basis of data fusion provided better results. Additionally, based on the changes and accuracy verification of the extracted built-up areas from 2012 to 2021, this study analyzed the accuracy and possible impact of NTL data and POI data in different stages of extracting urban built-up areas and obtained the application value of different data at different times. In combination with the actual development of the city, the feasible path for long time sequence monitoring in the built-up areas was deeply summarized by the study. In general, this study puts forward a feasible method for the long-term monitoring of built-up areas by NTL data and POI data, which improves the feasibility of long-term monitoring of built-up areas through the analysis of the influence of data in different development stages and periods. The fusion of NTL data and POI data can avoid the occasional influence of single data and has far-reaching practical significance for the formulation of urban long-term planning.

## Figures and Tables

**Table 1:** Study Data.

Data	Spatial Resolution	Data Sources	Access Time
Landsat7, Landsat8	30 m, 60 m	http://www.gscloud.cn/	1 May 2022
DMSP/OLS, NPP/VIIRS	1000 m, 500 m	https://eogdata.mines.edu/products/vnl/	1 May 2022
Amap	-	www.Amap.com	1 May 2022

**Table 2:** Statistical Yearbook Data of Built-up Areas in Kunming and Yuxi Region.

Kunming	Year	2012	2013	2014	2015	2016
	Aera (km^2^)	298.12	397.23	407.16	409.39	436.44
	Year	2017	2018	2019	2020	2021
	Aera (km^2^)	438.31	441.92	446.46	483.22	548.47
Yuxi	Year	2012	2013	2014	2015	2016
	Aera (km^2^)	24.11	24.56	33.57	37.14	38.12
	Year	2017	2018	2019	2020	2021
	Aera (km^2^)	38.47	38.69	39.25	42.19	46.2

**Table 3:** The Number of POI in Kunming and Yuxi from 2012 to 2021.

Kunming	Year	2012	2013	2014	2015	2016	2017	2018	2019	2020	2021
	POI Number	249,074	269,403	273,304	290,321	384,466	468,202	485,942	498,341	500,217	508,944
Yuxi	Year	2012	2013	2014	2015	2016	2017	2018	2019	2020	2021
	POI Number	163,089	170,372	177,635	183,897	194,482	308,719	347,648	367,741	369,013	374,538

**Table 4:** Verification Results.

NTL	2012		2013		2014		2015		2016	
	F1	Accuracy	F1	Accuracy	F1	Accuracy	F1	Accuracy	F1	Accuracy
	0.7233	88.02%	0.7245	87.98%	0.7331	88.19%	0.7318	88.67%	0.7238	88.45%
	2017		2018		2019		2020		2021	
	F1	Accuracy	F1	Accuracy	F1	Accuracy	F1	Accuracy	F1	Accuracy
	0.7401	88.89%	0.7307	89.02%	0.7297	89.31%	0.7301	86.22%	0.7406	85.89%
POI_NTL	2012		2013		2014		2015		2016	
	F1	Accuracy	F1	Accuracy	F1	Accuracy	F1	Accuracy	F1	Accuracy
	0.7891	91.78%	0.7862	92.24%	0.7902	92.13%	0.7845	92.10%	0.7913	92.18%
	2017		2018		2019		2020		2021	
	F1	Accuracy	F1	Accuracy	F1	Accuracy	F1	Accuracy	F1	Accuracy
	0.8502	95.01%	0.8471	94.49%	0.8549	95.31%	0.8122	93.22%	0.8204	93.18%

## Data Availability

Not applicable.
